# Succinimido–Ferrocidiphenol Complexed with Cyclodextrins Inhibits Glioblastoma Tumor Growth In Vitro and In Vivo without Noticeable Adverse Toxicity

**DOI:** 10.3390/molecules27144651

**Published:** 2022-07-21

**Authors:** Feten Najlaoui, Benoit Busser, Germain Sotoing Taïwe, Pascal Pigeon, Nathalie Sturm, Diane Giovannini, Naziha Marrakchi, Ali Rhouma, Gérard Jaouen, Stéphane Gibaud, Michel De Waard

**Affiliations:** 1 Laboratoire des Venins et Biomolécules Thérapeutiques LR11IPT08, Institut Pasteur de Tunis, 13, Place Pasteur, Tunis 1002, Tunisia; fatennajlaoui@yahoo.fr (F.N.); naziha.marrakchi@pasteur.rns.tn (N.M.); 2 Institute for Advanced Biosciences, INSERM U1209, CNRS UMR 5309, Université Grenoble Alpes, F-38000 Grenoble, France; bbusser@chu-grenoble.fr; 3 Research Unit of Plant Protection and Environment, Olive Tree Institute, BP 208, Tunis 1082, Tunisia; ali_rhouma@yahoo.com; 4 EA 3452/CITHEFOR, Université de Lorraine, 9 Avenue de la Forêt de Haye-BP 20199, CEDEX, F-54505 Vandoeuvre-lès-Nancy, France; 5 Department of Zoology and Animal Physiology, Faculty of Science, University of Buea, Buea P.O. Box 63, Cameroon; taiwe_sotoing@yahoo.fr; 6 Chimie ParisTech, 11 Rue Pierre et Marie Curie, CEDEX 05, F-75231 Paris, France; pascal.pigeon@chimieparistech.psl.eu (P.P.); gerard.jaouen@chimieparistech.psl.eu (G.J.); 7 Institut Parisien de Chimie Moléculaire (IPCM)–UMR 8232, Sorbonne Université, 4 Place Jussieu, CEDEX 05, F-75252 Paris, France; 8 Department of Pathology, Institute of Biology and Pathology, University Hospital of Grenoble, CS 10217, F-38043 Grenoble, France; nsturm@chu-grenoble.fr (N.S.); dgiovannini@chu-grenoble.fr (D.G.); 9 L’institut du Thorax, CNRS, INSERM, Nantes Université, F-44000 Nantes, France; michel.dewaard@univ-nantes.fr; 10 LabEx Ion Channels, Science and Therapeutics, F-06560 Valbonne, France

**Keywords:** ferrocenyl tamoxifen derivatives, cyclodextrin, glioblastoma, anticancer drug

## Abstract

SuccFerr *(N-[4-ferrocenyl,5-5-bis (4-hydroxyphenyl)-pent-4-enyl]-succinimide)* has remarkable antiproliferative effects in vitro, attributed to the formation of a stabilized quinone methide. The present article reports in vivo results for a possible preclinical study. SuccFerr is lipophilic and insoluble in water, so the development of a formulation to obviate this inconvenience was necessary. This was achieved by complexation with randomly methylated cyclodextrins (RAMEßCDs). This supramolecular water-soluble system allowed the in vivo experiments below to proceed. Application of SuccFerr on the glioblastoma cancer cell line U87 indicates that it affects the cellular cycle by inducing a blockade at G0/G1 phase, linked to apoptosis, and another one at the S phase, associated with senescence. Using healthy Fischer rats, we show that both intravenous and subcutaneous SuccFerr: RAMEßCD administration at 5 mg/kg lacks toxic effects on several organs. To reach lethality, doses higher than 200 mg/kg need to be administered. These results prompted us to perform an ectopic in vivo study at 1 mg/kg i.v. ferrocidiphenol SuccFerr using F98 cells xenografted in rats. Halting of cancer progression was observed after six days of injection, associated with an immunological defense response linked to the active principle. These results demonstrate that the properties of the selected ferrocidiphenol SuccFerr transfer successfully to in vivo conditions, leading to interesting therapeutic perspectives based on this chemistry.

## 1. Introduction

**Figure 1:**
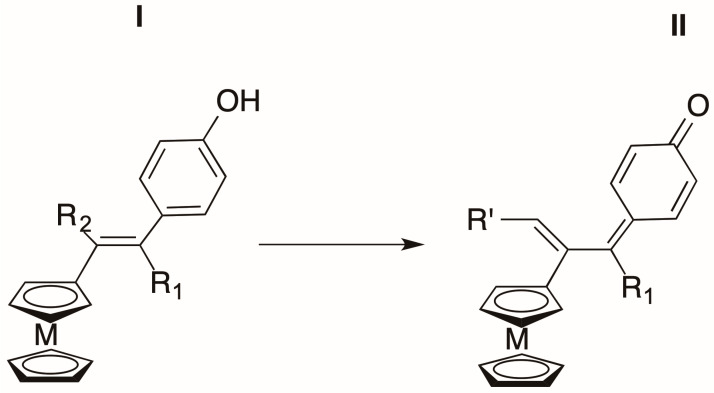
Access route via oxidation of ferrocene precursors to selectively electrophilic metallocene quinone methides. (**I**) Precursor of a metallocenic quinone methide inserted in a hydroxytamoxifen-like skeleton. (**II**) Example of organometallic and acyclic quinone methide obtained by oxidation of precursor (**I**) bearing a metallocenyl and a phenol group in trans configuration (with respect to the initial double bond). M = Fe; R1 = arene (preferentially -C_6_H_5_-*p*-OH); R2 = ethyl, (CH_2_)_3_X, with X = H, OH or succinimidyl; R’ = CH_3_, (CH_2_)_2_OH or (CH_2_)_2_-succinimidyl.

One of the stated aims of the bioorganometallic approach in medicinal chemistry is the bridging of gaps in the current suite of antitumoral metallodrugs that rely on the acid-base coordination chemistry of the Pt^2+^ complexes [1,2,3]. These metallodrugs, which primarily, but not exclusively, target the DNA bases [4,5], occur alone or in combination in more than 50% of cancer treatments [6]. This situation occurs despite the fact that metallodrugs have the following several drawbacks: (i) they create resistance problems, (ii) possess low selectivity between healthy and cancerous cells, (iii) give rise to high levels of toxicity and (iv) work in a relatively narrow therapeutic range. The development of bioorganometallic chemistry, a discipline defined by the existence of at least one direct covalent bond between a metal and a carbon atom (M-C), allows new approaches that possess several benefits, including the variety of metals (principally transition metals) available for use, an almost infinite number of accessible ligands, and the flexibility of these complexes in their reactivity that permits an intracellular multi-targeting approach to counter resistance [7]. To date, the metals that have yielded organometallic compounds with potential in biological applications are primarily Ir, Ti, Re, Tc, Pt, with substantial advances made for Ru and Au [8]. Meanwhile, iron, the most abundant transition metal in the human body, after some early challenges in the search for an organometallic approach compatible with biology [9], is now gradually working its way towards the top priorities. This effectively first became clear once ferrocene, the archetype of the metallocenes, came into use with the emergence of a new chemistry focused on electronic delocalization, supplanting the previous narrow focus on the oxidation of ferrocene to ferricenium, a compound that is unstable in aqueous media and has little biological activity [7,10,11]. In this context, the ferrocifens and ferrociphenols represent remarkable examples of biological efficacy, enabled by our understanding of their chemical mechanism of action in biological settings [9]. These tamoxifen derivative species are characterized by the central presence of the redox motif (ferrocenyl-ene-phenol), which by generating reactive oxygen species (ROS) on cancerous cells gives rise as its first metabolite to a novel functional group, an organometallic quinone methide (OM-QM, e.g., Figure 1).

Thanks to the electrophilic properties that can be modulated by the substituents involved, this entity can interact with nucleophilic proteins overexpressed in cancer cells but absent from healthy ones. The OM-QM group behaves as an amplifier of oxidative stress, enhancing antiproliferative effects and is a source of multitargeting capability [9]. However, recent research showed that for ferrociphenols to have anticancer activity, their ferrocenyl and phenol moieties should be trans in respect to the central double bond, as represented in Figure 1 for I, [12]. For this reason, diphenols (ferrocidiphenols: R1 = another *p*-hydroxyphenyl moiety), that always have this motif, since there are not Z/E mixtures, are compounds that are preferred over monophenols.

**Figure 2:**
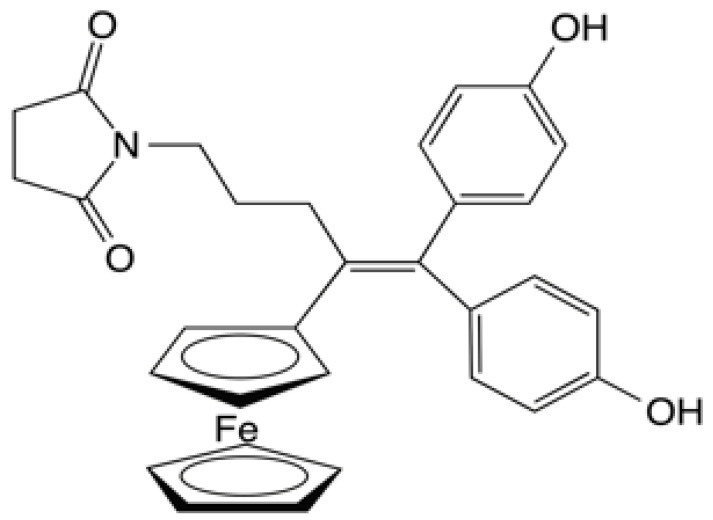
*N*-[4-ferrocenyl,5-5-bis (4-hydroxyphenyl)-pent-4-enyl]-succinimide (SuccFerr).

The ferrociphenols act via apoptosis (at ~10^−6^ M) and/or by senescence (at lower concentrations of ~10^−8^ M). The preference for one route over the other depends on the nature of the cancer cells and other parameters, such as concentration. They also act as inhibitors of cancerous stem cells, of thioredoxine reductase and in certain cases of cathepsin B. Of all the examples studied in vitro, the ferrociphenols bearing the substituent -(CH_2_)_3_–succinimidyl (e.g., SuccFerr, Figure 2) are particularly active on the panel of 60 cell lines of the NCI in Virginia [13]. The explanation for this exceptional activity has been attributed to a weak “lp…π” interaction, causing the intervention of a lone pair of a CO of the heterocycle and the π cloud of the quinone methide. The energy involved is very low, of the order of 1.5 kCal/mole. For more details, one can refer to the work of A. Vessieres et al., 2021 [14] as well as B. Sharma and V. Kumar, 2021 [15] and for the role of thioredoxin reductase as a pharmacological target, the work of G. Bjorkluund et al., 2021 [16].

The “lp…π” interaction had never before been reported in medicinal chemistry. It has the effect of stabilizing the organometallic quinone methide that is the highly active primary metabolite, while acting as a brake on the formation of competing secondary and counterproductive metabolites, as we have shown elsewhere [17,18]. However, it is not known whether this behavior retains its advantages in vivo if a new type of stable vehicle is used, rather than the fragile lipid nanocapsules (LNC) that are typically used with ferrocifens [19,20]. This is the question that is addressed herein within the framework of the preclinical trial we developed. The trial is instituted on the use of a new vehicle of the randomly methylated cyclodextrin (RAMEßCD) type to form a novel, water soluble supramolecular complex in a CD:SuccFerr ratio of 2:1 [21] and in a cellular cycle study with glioblastomas, illustrating a blockage in the S phase related to senescence and another in the Go/G1 phase linked to apoptosis. These initial steps were followed by a toxicology study of the complex on Fischer rats to define the lethal dose and the possible toxicity affecting a number of organs. Finally, a proof-of-concept study was performed on the anti-tumor properties of the complex at a dose of 1 mg/kg, using a xenografted very aggressive F98-type glioblastoma cell line. The anti-tumor dose used was shown to be highly effective and substantially lower than the doses yielding toxicity, demonstrating the preclinical value of the complex, thereby meeting our initial objectives.

Very recent studies in fifteen molecularly diverse glioblastoma patient-derived cell lines (PDCLs) also showed an unexpected behavior of SuccFerr (also known as P722) in comparison to five other tested ferrociphenol derivatives, with the highest and lower IC_50_ of the six compounds (from 10 nM to ≈ 30 µM, IC50 ratio ≈ 3000) [22]. This suggests that the response of a future treatment with SuccFerr could be very dependent on the patient.

Finally, cyclodextrins should not be considered as simple solubility enhancers, since the formation of the inclusion complex might also affect the bioavailability of the tissue distribution of drugs, and thereby therapeutic response [23,24].

## 2. Results

### 2.1. Physicochemical Characterization of the Freeze-Dried SuccFerr Complex

Lyophilized samples (frozen overnight at −20 °C and freeze-dried in a SMH15 freeze-drier, Usifroid, Maurepas, France) were tested by DSC and XRD to confirm the complexation on solid-state samples. The concentration of SuccFerr, determined by HPLC, was 5 mg/g.

**Figure 3:**
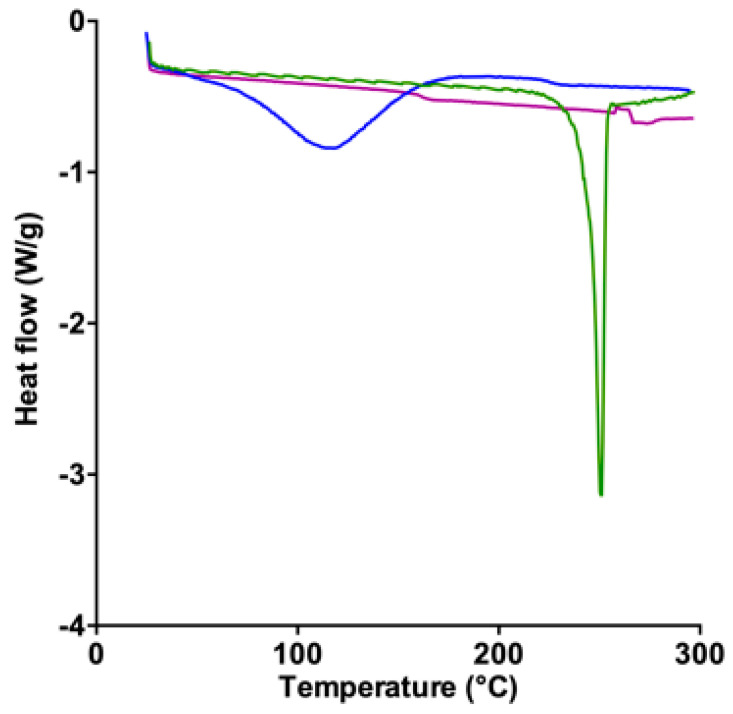
DSC curves for SuccFerr (green), SuccFerr:RAMEßCD (purple) and RAMEßCD (blue).

The DSC profile of pure RAMEβCD (Figure 3) exhibited a large endothermic event between 90 and 140 °C, attributed to the evaporation of the absorbed water, whereas the melting point of pure SuccFerr occurred at 251 °C. Besides this, the thermogram of SuccFerr:RAMEβCD does not exhibit any endothermic event, suggesting a real association of SuccFerr and the RAMEßCD.

**Figure 4:**
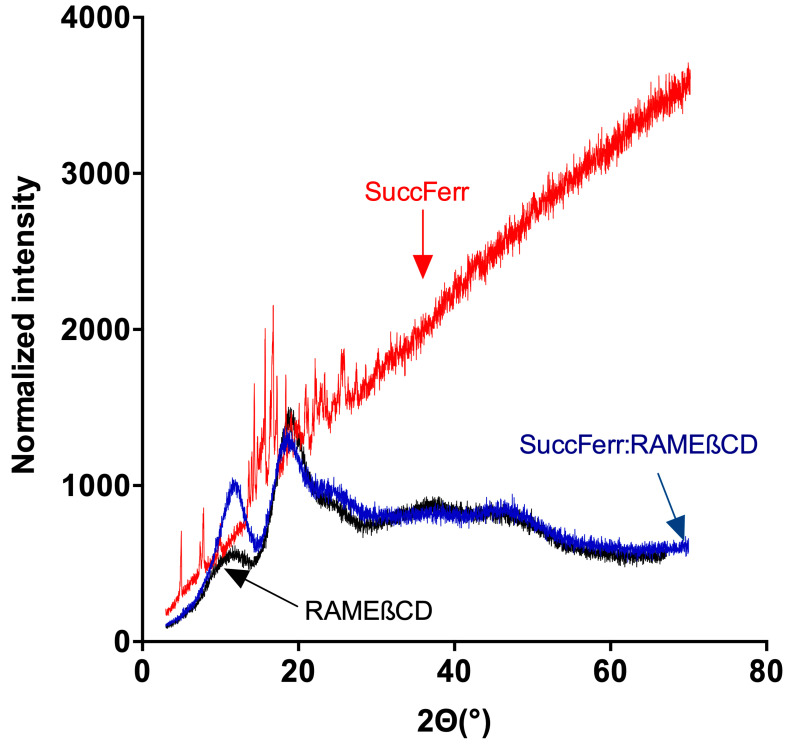
XRD diffraction spectra of SuccFerr, SuccFerr:RAMEßCD and RAMEßCD.

The XRD diffractogram (Figure 4) of pure SuccFerr exhibited numerous peaks, characteristic of its crystalline form. On the contrary, RAMEβCD showed only a broad amorphous band. The diffraction pattern of the freeze-dried complex showed that the drug had lost its crystalline state. This phenomenon is usually attributed to the complexation of the drug [25,26].

### 2.2. Pharmaceutical Properties

#### 2.2.1. Release Profile of SuccFerr:RAMEßCD Complex

**Figure 5:**
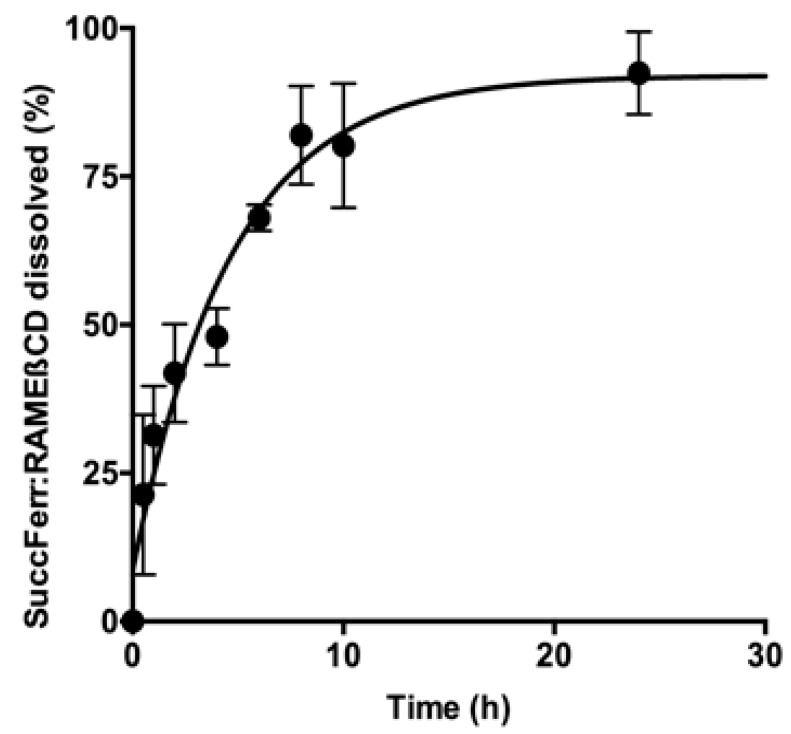
Dialysis of SuccFerr:RAMEßCD in a dialysis bag (final concentration: 5 mg/mL)—cutoff 14,000 Da.

Since the aim of this work was to prepare a soluble formulation of SuccFerr, the release profile is a key parameter. An immediate and complete dissolution of SuccFerr:RAMEßCD was observed in distilled water. Nevertheless, pure SuccFerr cannot be dissolved and was undetectable. A second experiment was carried out in a dialysis bag to consider the diffusion of RAMEßCD through the dialysis membrane. The profile of the curve displayed in Figure 5 shows that there is more than 80% in the external phase after 10 h.

#### 2.2.2. Anticancer Activity

**Figure 6:**
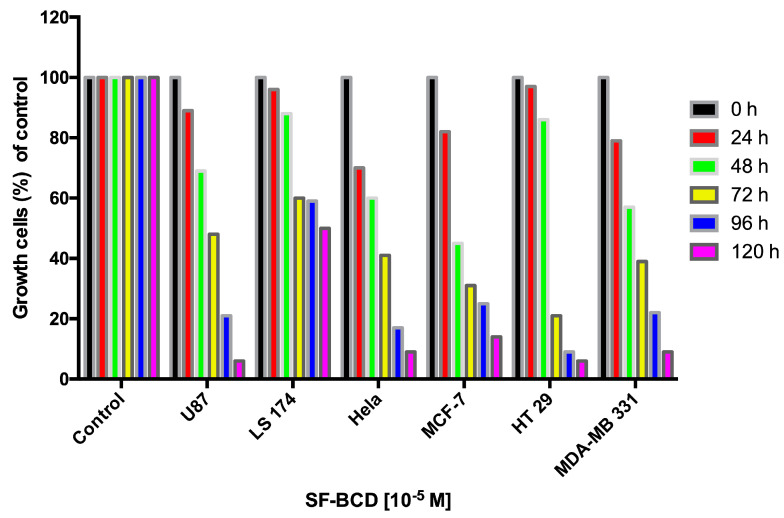
Inhibition of cell growth followed during 5 days by 10^−5^ M of complexed SuccFerr (SuccFerr:RAMEβCD) for various cancer cell lines (U87, LS174, Hela, MCF-7, HT29, MDA-MB231).

*SuccFerr:RAMEβCD activity in cancer cells*. The global anticancer activity of the SuccFerr:RAMEβCD molecule was assessed by its impact on cell growth (average of three assays) using several cancer cell lines (Figure 6). The highest efficacies of SuccFerr:RAMEβCD were evident on U87 gliobastoma cells, Hela cells, the breast tumor cell lines MCF-7 and MDA-MB 231, and HT29 cells. Since we were interested in glioblastoma cancers, we focused therein on U87 cells and extended our analyses also to F98 glioblastoma cells. A first assessment of U87 glioblastoma cells provided an encouraging half-inhibiting concentration (IC_50_) of 6.2 × 10^−7^ M (CI95%, (3.8 × 10^−7^; 1.0 × 10^−6^)). Therefore, we decided to expand our study on the potential of SuccFerr:RAMEβCD for inhibiting glioblastoma cells in all the following experiments.

**Figure 7:**
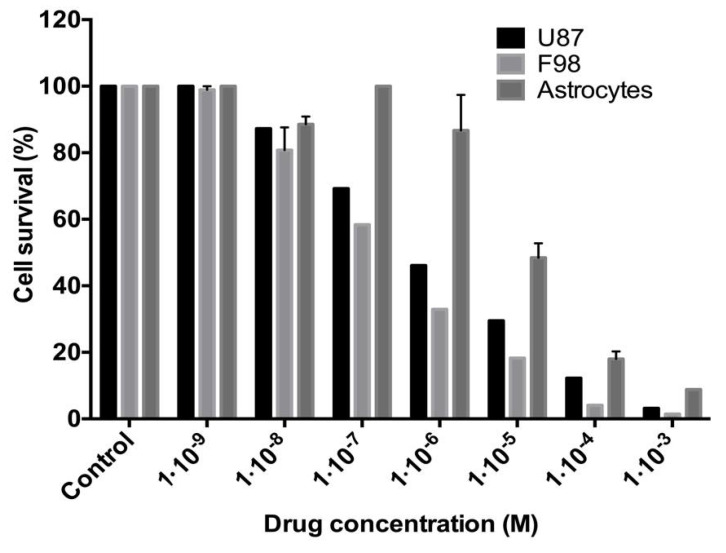
Cytotoxity of SuccFerr:RAMEβCD on U87 and F98 glioma cell lines and on astrocytes, as measured by cell survival percentage. Incubation: 72 h at 37 °C.

*SuccFerr:RAMEβCD activity on astrocytes and glioblastoma cells.* The cytotoxicity of SuccFerr:RAMEβCD was further evaluated on the survival of non-cancerous astrocytes and on the F98 glioblastoma cell line (Figure 7). Importantly, we noticed that SuccFerr:RAMEβCD produces a relatively low toxicity on astrocytes (IC_50_ = 8.3 × 10^−6^ M; CI95% (5.7 × 10^−6^; 1.2 × 10^−5^)), compared to the effect on the human U87 glioblastoma cell line (IC_50_ = 6.2 × 10^−7^ M; (CI95%, (3.8 × 10^−7^; 1.0 × 10^−6^)). With the aim to perform further in vivo investigations onto xenografted rats, the cytotoxicity of SuccFerr:RAMEβCD was also assessed on murine F98 glioblastoma cell survival and yielded results quite similar to those on U87 cells (IC_50_ = 1.6 × 10^−7^ M; CI95% (1.0 × 10^−7^; 2.4 × 10^−7^)).

**Figure 8:**
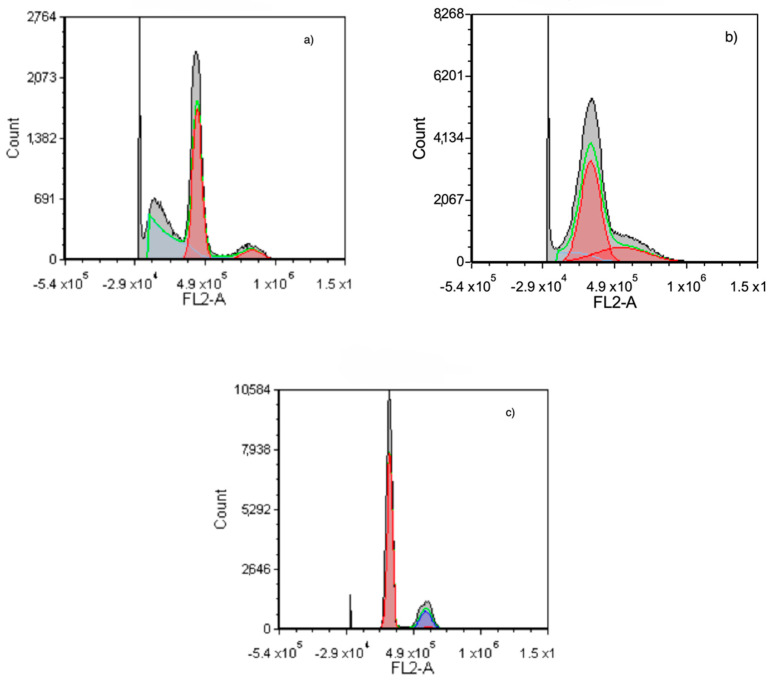
Flow cytometry analysis of DNA content for cell cycle analysis of U87. Cells were treated with: (**a**) 10^−5^ M of SuccFerr, (**b**) 10^−5^ M of complexed SuccFerr (SuccFerr:RAMEβCD), and (**c**) blank formulation (RAMEßCD). Modeling of the cell cycle was carried out using a mathematical tool (MultiCycleAV DNA, FCS Express 5 from De Novo software, Pasadena, CA, USA). DNA content was measured by propidium iodide staining.

**Figure 9:**
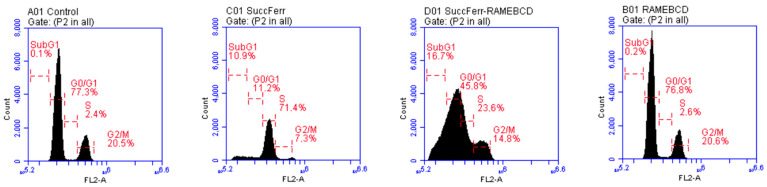
Distribution of cells in different phases of the cell cycle derived from flow cytometry on U87 cells. Cells were treated with: 10^−5^ M of SuccFerr, 10^−5^ M of complexed SuccFerr (SuccFerr:RAMEβCD) or blank formulation (RAMEßCD). Control = no treatment.

*SuccFerr:RAMEβCD activity on glioblastoma cell cycle*. We also assessed the in vitro activity of SuccFerr:RAMEβCD on the U87 cell cycle (Figure 8). The RAMEβCD (Figure 8c) did not modify the cell cycle distribution, since it looked similar to the non-treated control cells. The SuccFerr moiety alone induced a strong blockade of the cell cycle in the synthesis (S) phase, as well as in the sub-G1 Phase (Figure 8b and Figure 9). The number of cells in the S increased from 2.4% (controls) to 71.4% when cells were treated with SuccFerr (Figure 9). Additionally, the number of cells in the sub-G1 phase increased from 0.1% (controls) to 10.9% when the cells were treated with SuccFerr (Figure 9). Similarly, the complexed SuccFerr:RAMEβCD severely altered the cell cycle distribution (Figure 8a). The treatment induced a major increase in the sub-G1 population, which is commonly considered for apoptotic cells. However, the S phase blockade also increased, as already obtained with the SuccFerr compound alone. The precise quantification of cells within each phase of the cell cycle is detailed in Figure 9.

**Figure 10:**
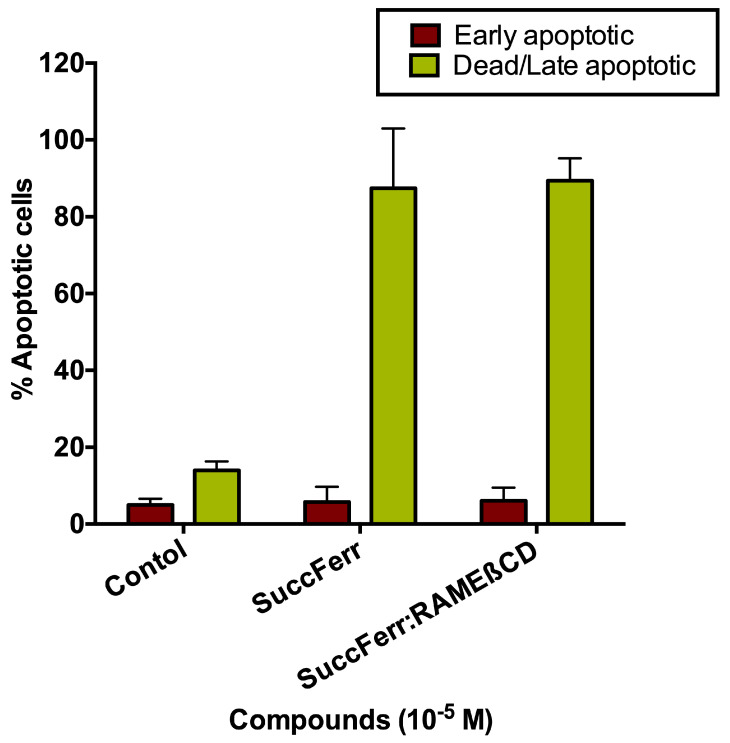
Percentage of apoptotic U87 cells determined by flow cytometry: annexin V assay for early apoptosis and caspase-3 assay for late apoptosis. Control is for the RAMEβCD condition.

The number of cells in the sub-G1 phase increased from 0.1% (controls) to 16.7% when cells were treated with SuccFerr:RAMEβCD (Figure 9), suggesting an important apoptotic phenomenon that will be characterized and quantified specifically (Figure 10). When cells were treated with SuccFerr:RAMEβCD, the number of cells in the S increased from 2.4% (controls) to 23.6% (Figure 9).

*SuccFerr: RAMEβCD-induced cell apoptosis*. Because the SuccFerr and SuccFerr:RAMEβCD compounds induced an increase in the sub-G1 phase, apoptosis induction was quantified with an activated-caspase-3 assay and an annexin V assay. Cells were treated with annexin V and the mixture was then analyzed using a flow cytometer to obtain two-scattered plots. Cells that bind to annexin V are at the early stages of cell death. Another assay was run with caspase-3 to look for the late stages of apoptosis. The percentage of cells that underwent apoptosis was determined for each experiment, as shown on Figure 10.

Clearly, based on these data, we show that all SuccFerr formulations induced an increase in apoptotic cell signal compared to the RAMEβCD controls. The non-treated cells behaved in a similar way to the RAMEβCD condition (not shown).

#### 2.2.3. Hemolytic Activity

**Figure 11:**
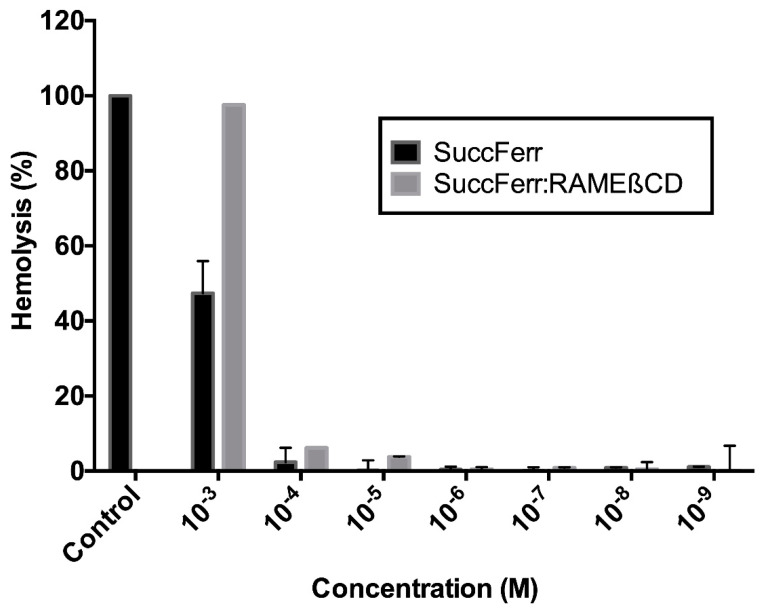
Hemolysis properties of SuccFerr and SuccFerr:RAMEßCD after 1 h incubation doses ranging from 10^−3^ to 10^−9^ M. Data are expressed in percentage of hemolysis compared to the positive control (100%). Triton X-100 (1%) was used as positive control.

Methylated cyclodextrins are known to have hemolytic properties [27]. It was, therefore, necessary to compare the hemolytic properties of the free drug with the complex before envisioning any form of in vivo injection. In our experiment, SuccFerr:RAMEßCD was slightly more hemolytic than SuccFerr itself at 10^−3^ M (Figure 11). However, this hemolytic activity was greatly reduced at 10^−4^ M and at 10^−5^ M and absent at 10^−6^ M. These results indicate that there is a considerable concentration margin in terms of safety of intravenous administration, considering an IC_50_ value in the submicromolar range (1.6 × 10^−7^ M; CI 95% (1.0 × 10^−7^; 2.4 × 10^−7^).

### 2.3. Animal Study

#### 2.3.1. Acute Toxicity Study of SuccFerr:RAMEβCD Administered Intravenously or Subcutaneously

The general behavioral changes in the rats were observed following intravenous administration of SuccFerr:RAMEβCD at 25, 50, 100, 200, 400, 800, 1600, 3200 and 6400 mg/kg doses, which were graded through time. The administration of SuccFerr:RAMEβCD at doses lower than 100 mg/kg in rats did not produce any abnormality in fur or eye color, nor asthenia, anorexia, salivation, piloerection, change in locomotor activity or diarrhea in any of the treated rats. In addition, no animal deaths were recorded on the days after administration. The results indicated that SuccFerr:RAMEβCD acute treatment by the intravenous route at doses up to 100 mg/kg also did not produce any sign of toxicity or death during 14 days of observation. While doses of 25, 50 and 100 mg/kg did not cause any detectable changes, a dose of 200 mg/kg seemed to be lethal and caused three deaths out of ten rats within 24–36 h. Signs and symptoms, which occurred in response to SuccFerr:RAMEβCD, included a decrease in motor activity and respiration. The rats that died from a high dose (from 200 mg/kg to 6400 mg/kg) of SuccFerr:RAMEβCD showed signs of respiratory failure (decreased respiratory rate and irregular breathing) before death. The internal organs of both controlled and treated groups did not show any unusual signs and were found to be normal in both size and color. The lethal dose (LD_50_) of SuccFerr:RAMEβCD in rats was determined to be 251.74 mg/kg (Table 1).

There were no deaths, or any signs of toxicity observed after subcutaneous administration of single doses of the SuccFerr:RAMEβCD at any dose level up to 200 mg/kg. However, at doses starting at 400 mg/kg, SuccFerr:RAMEβCD caused slow movement of the animal, decrease in aggressiveness, respiratory distress, and pain sensibility. It also produced a significant change in general behavior, breathing, cutaneous effects, sensory nervous system responses, gastrointestinal effects and mortality in male and female rats. The intensity of the responses grew with increasing doses and the effects of SuccFerr:RAMEβCD persisted for more than 2 h after subcutaneous administration. The rats that died from a high dose (400 mg/kg) of SuccFerr:RAMEβCD showed signs of respiratory failure (decreased respiratory rate and irregular breathing) before death. The lethal dose (LD_50_) obtained for the measurement of the acute subcutaneous toxicity of SuccFerr:RAMEβCD in rats was 384.52 mg/kg (Table 1).

#### 2.3.2. Body and Organ Weight: Maximum Tolerated Dose (MTD)

**Figure 12:**
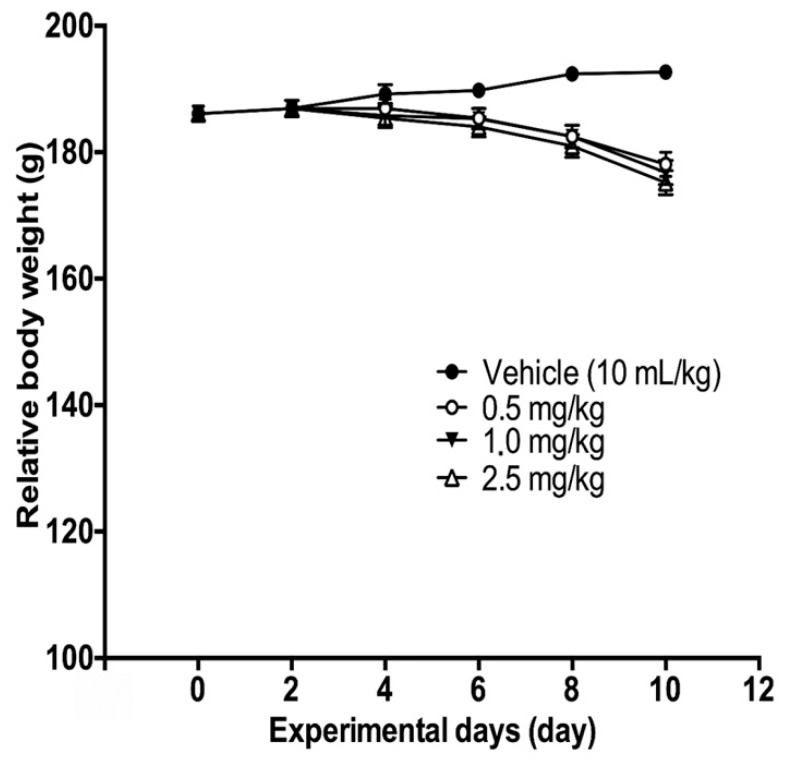
Relative weight of control rats and rats treated intravenously with SuccFerr:RAMEβCD (0.5; 1; 2.5 mg/kg) for 10 days.

As shown in Figure 12, both control and animals treated with NaCl 0.9% presented constant increases in body weight. In contrast, animals receiving SuccFerr:RAMEβCD intravenously (0, 1.0 or 2.5 mg/kg) showed decreased body weight at day 10, as compared to control rats. This effect was, however, not statistically significant (*p* > 0.05). Macroscopic analyses of target organs of treated animals (liver, heart, lung, kidney, and spleen) did not show significant changes in color and texture when compared with the control group. Relative weights of organs were not significantly affected by SuccFerr:RAMEβCD treatment (Table 2).

#### 2.3.3. Hematological and Biochemical Parameters

Intravenous administration of SuccFerr:RAMEβCD to rats on every second day (48 ± 2 h) and for 10 days did not cause any significant change in the hematological profile compared to a control group (Table 3). In addition, SuccFerr:RAMEβCD administered intravenously did not cause any significant change in serum glucose, albumin, LDL or HDL. However, liver enzyme levels (AST, ALT and ALP) increased in a dose-dependent manner. Serum total protein content, triglyceride and total cholesterol levels did not significantly change (*p* < 0.05) (Table 4).

Blood concentration of creatinine, urea, uric acid, Cl^−^, Ca^2+^ and inorganic phosphorus did not significantly decrease in SuccFerr:RAMEβCD-treated animals as compared to the control group (Table 5). Indeed, as shown, there were no significant changes in the daily urine excretion of creatinine, protein, urea, Na^+^, K^+^, Cl^−^, Ca^2+^, Mg^2+^ or inorganic phosphorus, even at the dose of 1.5 mg/kg SuccFerr:RAMEβCD.

#### 2.3.4. In Vivo Evaluation of SuccFerr:RAMEβCD Anticancer Activity in a Murine Glioblastoma Model

**Figure 13:**
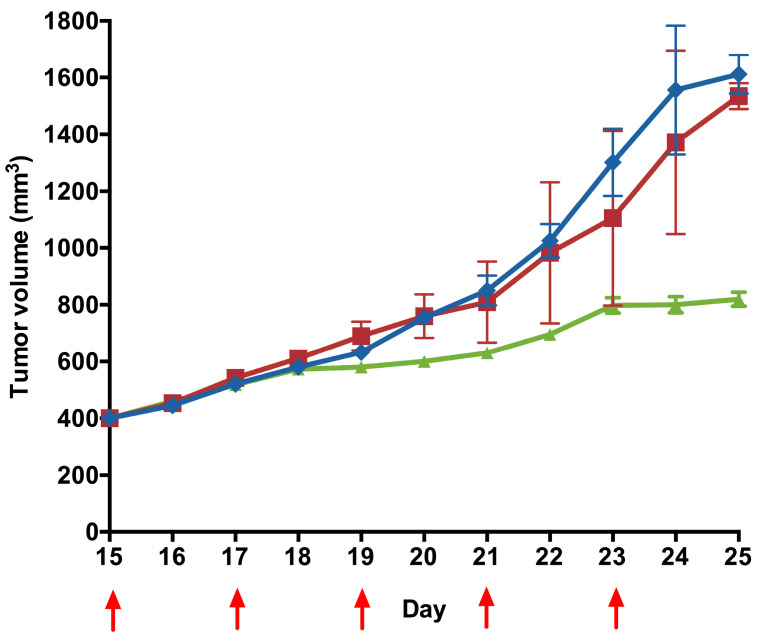
In vivo antitumor efficacy. Tumor volume after injection of 1 mg/kg of SuccFerr:RAMEßCD (-▲-), of the equivalent dose of RAMEßCD (-■-) and of NaCl (0.9%; 10 mL/kg; -♦-). n = 8. Mean tumor volumes ± SD. Treatment days are indicated by red arrows.

The F98 murine heterotopic glioblastoma model was used to evaluate the efficacy of SuccFerr:RAMEβCD in vivo. The F98 tumors were grown subcutaneously for 15 days to reach a mean of 400 mm^3^ volume. These aggressive tumors were treated by either vehicle (NaCl 0.9%), RAMEβCD alone, or SuccFerr:RAMEβCD (1 mg/kg) by i.v. injections, every 2 days during 10 days. The tumor volumes of the control group grew quickly and reached the maximal ethical tumor volume within 10 days (Figure 13). On the contrary, SuccFerr:RAMEβCD-treated tumors almost stopped their growth after the onset of the treatment. The differences between tumor volumes of the treated and control groups were statistically different 6 days after treatment initiation. The pathological examination performed at the end of the experiment found all the livers and kidneys to have a normal histological appearance.

#### 2.3.5. Histological Analyses

Considering the aggressive protocol involving repeated injections of SuccFerr:RAMEβCD, it seems necessary to take an interest in the condition of the rat livers and kidneys. Specimen sections of the control groups, and of SuccFerr:RAMEβCD and blank RAMEβCD-treated groups showed normal histological appearances of these organs, meaning that no visible damage manifestation was inflicted by SuccFerr-RAMEβCD prolonged treatment (Supplementary Figure S1).

## 3. Discussion

Despite extensive efforts in research, the prognosis of patients with glioblastoma remains poor. By the time a glioma becomes symptomatic, it is almost always too late in its biological course and treatment remains ineffective. The treatment of glioblastoma is still considered as a major unmet need in the oncology field.

In this work, we studied a supramolecular assembly (SuccFerr:RAMEβCD); the host molecule is a cyclodextrin (RAMEβCD) and the guest molecule (SuccFerr (*N-[4-ferrocenyl,5-5-bis (4-hydroxyphenyl)-pent-4-enyl]-succinimide*)) is known to have a remarkable antiproliferative effect in vitro. In fact, the grafting of a ferrocenyl organometallic group onto a phenolic compound was first performed to increase cytotoxicity [28]. Among all the molecules called ferrocifens, the *4-ferrocenyl-5,5-bis-(4-hydroxy-phenyl)-pent-4-enyl* structure has led to a series of compounds [26]. Interestingly, the most active compounds also have a phthalimide group, a succimide group or an -OH group, explaining our choice in this study.

Nevertheless, the low solubility of these lipophilic compounds requires specific pharmaceutical development. These ferrocidiphenol have, for example, been encapsulated in PEG-PLA nanoparticles [29] and in lipid nanocapsules [30], which allowed in vivo administration. In addition, an in vivo study recently demonstrated improved survival (orthotopic melanoma tumors) and slower tumor growth with SuccFerr-loaded LNC (intraperitoneal injection) compared to anti-CTLA4 mAb [31].

In this study, SuccFerr was included in methylated-β-cyclodextrin (RAMEßCD), tested on different cell lines (U87, LS174, Hela, MCF-7, HT29, MDA-MB231) and finally evaluated in vivo on Fischer male rats.

The complexation of SuccFerr with RAMEßCD was previously studied by phase solubility experiments and molecular modelling [21], demonstrating that major systems in solution are 2-CD systems. Moreover, succinimidylpropyl is favorably included by the large side and the ferrocenyl (Fc) is favorably included by the narrow side [21]. In this paper, a variety of spectroscopic techniques were used for the solid-state characterization. DSC thermograms of each two raw materials, compared to the thermogram of the freeze-dried SuccFerr:RAMEβCD, ascertained that there is a real inclusion of the SuccFerr into the RAMEβCD due to the absence of both melting endotherms in the DSC thermogram of the complex. In addition, XRD demonstrated the formation of an amorphous inclusion complex between RAMEβCD and SuccFerr. The diffractogram of pure SuccFerr revealed numerous peaks with high and rising background compared to SuccFerr:RAMEßCD. Hence, SuccFerr is present but it has lost crystalline and amorphous geometry in the complex. Dissolution study was carried out in water and an immediate dissolution of SuccFerr:RAMEßCD was observed (100%). However, pure SuccFerr cannot be dissolved and was not detectable. Next, we analyzed the performance of dissolution of the complex realized in a dialysis bag. The release profile of SuccFerr:RAMEßCD suggested that the dialysis through the membrane was quite slow, but more than 70% was in the external phase after 8 h. SuccFerr:RAMEßCD was tested in vitro in survival assay on F98 glioma cells and demonstrated a strong effect with an IC_50_ of 1.6 × 10^−7^ M (CI95% (1.0 × 10^−7^; 2.4 × 10^−7^)). In comparison to ansaFcdiOH (IC_50_ = 10^−7^ M), an analogous drug, which has already demonstrated promising activity on brain tumors [32], SuccFerr:RAMEßCD displayed a quite similar IC_50_ value on F98 cell lines, demonstrating its hopeful potent activity.

In order to obtain a deeper insight into the SuccFerr:RAMEßCD mechanism of action, cell cycle and apoptotic analyses were performed. As result, SuccFerr:RAMEßCD altered the F98 cell cycle with arrest in S phase and in G0/G1, with associated apoptosis induction. The arrest in S phase with ferrocenyl tamoxifen derivatives has already been described [29] and a senescence has also been studied [33]. In this article, the cellular cycle study illustrates a blockage in the S phase related to senescence and in the G0/G1 phase linked to apoptosis. The accurate molecular mechanism behind the senescence is not known and further studies will help to investigate the underlying mechanism. Nevertheless, several hypotheses have been discussed by C. Bruyère et al. [33].

Based on clinical settings, we planned, for the in vivo efficacy study, a treatment protocol composed of repetitive doses.

Maximum tolerated doses were determined to set the doses of animal experiments. In the acute toxicity study, no adverse effect was observed up to the doses of 100 and 200 mg/kg of SuccFerr:RAMEßCD, respectively administered by the intravenous route. Rats receiving this complex at the doses between 200 and 800 mg/kg via the intravenous route showed hypoactivity, marked by a reduction in aggressiveness, locomotion and pain sensitivity. It can be thought that this complex possesses depressive effects on the nervous system. This may justify the reduction in pain sensibility observed in the treated animals. All the animals treated survived beyond the 14 days observation period. It was observed that the adverse effects of higher doses appear 2 h after administration and that the disappearance of these signs was dose related. The median acute toxicity value (LD_50_) administered intravenously or subcutaneously is then above 251.74 or 384.52 mg/kg body weight, respectively.

Sub-acute treatment showed that intravenous administration on every second day (48 ± 2 h) for 10 days did not produce any death or clinical signs of toxicity. There was no significant change in the relative body and organ weight of rats treated (0.5–1.5 mg/kg). This suggests no glossy toxic effect. It has been established that the highest overall concordance of toxicity in animals with humans concerns hematological parameters. Analyses of the blood parameters are relevant to risk evaluation, as the changes in the hematological system have a higher predictive value for human toxicity, when the data are translated from animal studies [34]. Therefore, it can be concluded that SuccFerr:RAMEßCD had no adverse effects on the hematological parameters of the rats [35]. A non-significant increase in the number of white blood cells in the rats treated with the dose of 0.5 mg/kg directly indicates the strengthening of the organism’s defense [36,37]. This elevation in total leucocyte count suggests that this complex contains biologically active principles that have the ability to boost the immune system through increasing the population of defensive white blood cells. Although not significant, a global increase was observed in red blood cell count, hematocrit, hemoglobin concentration and platelets, implying that there may be a possible increase in erythropoiesis with increasing doses.

There were no adverse effects on the usual markers of liver and kidney toxicity, i.e., serum levels of ALT/AST and creatinine, respectively. Furthermore, there were no changes in kidney levels of urea, uric acid, Na^+^, K^+^, Cl^−^, Ca^2+^, Mg^2+^ or inorganic phosphorus. These results, associated with the observed normal creatinine levels, suggest that the intravenous administration did not alter kidney function at the doses studied [38,39]. No difference was observed in the weight or structure of the liver, kidney, lung and pancreas between the control and the treated groups. Altogether, the sub-acute study indicates that administration did not induce detrimental changes or morphological alterations in these organs.

## 4. Materials and Methods

### 4.1. Materials

Randomly methylated-β-cyclodextrin (1.6–2.0 methyl unit per anhydroglucose unit; RAMEβCD) was purchased from Sigma-Aldrich (Saint-Quentin-Fallavier, France). All other reagents were analytical grade from either Merck Eurolab (Fontenay-sous-Bois, France) or Acros organics (Noisy-le-Grand, France) and were used as received.

### 4.2. Synthesis and Quantification of Succinimido–Ferrocidiphenol (SuccFerr)

#### 4.2.1. Chemical Synthesis of SuccFerr

SuccFerr was prepared in three steps, as previously described [40]. Firstly, the chlorinated ketone was synthesized by the Friedel–Crafts reaction. Secondly, the chlorinated alkene was prepared by the McMurry coupling reaction. Finally, the imide ferrocidiphenol was obtained by substitution of the chlorine atom by the succimide. The compound purity was greater than 95%, as confirmed by HPLC.

#### 4.2.2. Quantification of SuccFerr

Determination of the ferrocidiphenol was carried out by high performance liquid chromatography (HPLC) or by UV–vis spectrophotometry, after dissociation of the complex by an initial dilution (1%) with pure dimethylsulfoxide (DMSO). The appropriate final adjustments were carried out with distilled water. For HPLC determinations, a 20 µL sample was injected into a C_18_ column (5 µm, 4.6 mm i.d. × 25 cm, Macherey-Nagel, Eckbolsheim, France) using an autosampler (WISP 712, Waters, Guyancourt, France). The mobile phase was a mixture of acetonitrile and water (65/35, *v/v*) at a flow rate of 1.5 mL/min (SP8800 pump, Spectra Physics, Milpitas, CA, USA). Detection was performed with a UV spectrophotometer at 286 nm (Waters 490E detector) using a SP-800 integrator (Spectra Physics). The limit of detection (LOD) was 2.3 mg/L and the limit of quantification (LOQ) was 7.6 mg/L. Standard curves were drawn between 7.8 and 125 mg/L (r > 0.99).

### 4.3. Solid Phase Analysis of Succ:RAMEβCD

The complexation of SuccFerr with RAMEßCD was previously studied by phase solubility experiments [21]. The curves exhibit positive curvature, described as Ap-type following Higuchi and Connors’ classification [41]. This is observed if more than one cyclodextrin can complex the drug corresponding to 1:2, 1:3, 1:4 (or more) stoichiometries. Molecular modeling has also demonstrated that major systems in solution are 2-CD systems. Moreover, succinimidylpropyl is favorably included by the large side and the ferrocenyl (Fc) is favorably included by the narrow side [21]. In this paper, we focused on the pharmaceutical aspects of the freeze-dried Succ:RAMEβCD complex.

To obtain a lyophilized sample, an excess of SuccFerr (30 mg) was stirred (25 °C; 24 h) in distilled water (2 mL) containing 160 mM of RAMEβCD and filtered through a 0.22 mm membrane filter (Millipore HA). The filtrate was freeze-dried and stored at +4 °C until use. The complexation efficiency of SuccFerr was determined by HPLC, as previously described. The solid phase analysis of the complexes was performed on these lyophilized samples.

#### 4.3.1. X-ray Diffraction (XRD)

The PXRD measurements were performed using a PanalyticalX’Pert Pro diffractometer equipped with a Cu tube, a Ge(111) incident-beam monochromator (l = 1.5406 Å) and an X’Celerator detector. Data collection was carried out in the scattering angle range 3–70, with a 0.0167 step over 90 min.

#### 4.3.2. Differential Scanning Calorimeter (DSC)

The DSC thermograms were obtained on a scanning calorimeter (DSC Q10, TA Instruments). The instrument was calibrated at various temperatures before starting the procedure. Each sample weighed 5 mg and was heated in sealed aluminum pans under a nitrogen environment at 50 mL/min. The limit of detection was controlled from 10 °C to 300 °C and the ramp was 10 C/min.

### 4.4. Pharmaceutical Properties of the SuccFerr:RAMEßCD Complex

#### 4.4.1. Dissolution and Diffusivity across a Dialysis Membrane

To investigate the dissolution, an amount (5 mg) of the lyophilized SuccFerr:RAMEßCD complex was suspended in water. The suspension was incubated at 25 °C in a shaking-bath (200 strokes/min, Heito, France). One-milliliter aliquots were taken at various time intervals up to 24 h and centrifuged× 7200*g*, 5 min, (Denver Instruments, Bohemia, NY, USA). Each supernatant was analyzed by HPLC to assess the drug contents. The results are presented as mean ± SD of triplicate experiments. A dialysis was also realized under magnetic stirring with a 14,000 Da cut-off bag, containing 2 mL of an aqueous solution of 5 mg of SuccFerr:RAMEßCD and 498 mL of distilled water. Aliquots were taken and analyzed as previously described.

#### 4.4.2. Cell Culture Experiments

For all cell treatments, the molecules of interest were added simultaneously with fresh medium during the mentioned period of time, without ever reaching cellular confluence. All cell lines were commercially available and purchased from ATCC.

#### 4.4.3. In Vitro Cytotoxicity on Glioblastoma U87 and F98 Cells in Culture

A preliminary experiment has been carried out on various commercial cell lines (U87, LS174, Hela, MCF-7, HT29, MDA-MB231) during 5 days, with a concentration of 10^−5^ M of complexed SuccFerr (SuccFerr:RAMEßCD). Subsequently, cytotoxic activities of SuccFerr and the SuccFerr:RAMEßCD complex were tested on glioblastoma cell lines (U87 and F98). The concentrations of SuccFerr and SuccFerr:RAMEßCD required to induce a decrease in cell viability were assessed by determining the ability of the U87 and F98 cells to reduce the tetrazolium dye in the MTT assay. The assay is based on the reduction by viable cells of the soluble yellow MTT by mitochondrial succinate dehydrogenase to a colored formazan complex, which is quantified spectrophotometrically at 560 nm [42]. The U87 or F98 cell suspensions were seeded in 96-well culture plates at a density of 5 × 10^3^ cells per well. After 12 h, varying amounts of SuccFerr and SuccFerr:RAMEßCD, ranging from 10^−3^ to 10^−9^ M, were added to the wells, and the culture plates were incubated for 72 h at 37 °C. For the MTT assay, 100 µL of MTT (5 mg/mL stock solution in phosphate-buffered saline, PBS) was added to each well and cells were incubated for 3 h at 37 °C. A 100 µL volume of dimethylsulfoxide (DMSO) was added to dissolve the formazan crystals. The optical density was measured at 560 nm with a microplate reader. The absorbance is proportional to the viable cell number, and survival was calculated as the percentage of the staining values of the untreated cultures. The IC_50_ were calculated via nonlinear regression using GraphPad Prism 6.0. (Y = Bottom + (Top-Bottom)/(1 + 10^((LogIC_50_-X) × HillSlope))).

#### 4.4.4. Cell Cycle Analysis

A suspension of 5 × 10^5^ U87 cells was seeded in 6-well culture plates with 2 mL of medium per well. After 12 h, the culture media was removed and cells were treated with 10^−5^ M of SuccFerr (as free SuccFerr or SuccFerr:RAMEβCD) and blank formulation (RAMEßCD); the culture plates were incubated for 96 h at 37 °C. At the end of the experiment, the cells were trypsinized, washed twice with PBS (without Ca^2+^ and Mg^2+^) and incubated with cold ethanol overnight at 4 °C. Cells were then centrifuged, washed one time with PBS and then incubated with 0.5 mL PI/RNase (30 min, in dark). The cell cycle was analyzed with an Accuri C6 flow cytometer (BD Biosciences) in at least three experiments, using a total of 50,000 cells in each sample. Data were analyzed by FCS express 5 (De novo software, USA).

#### 4.4.5. Cell Apoptosis Assay

Cell apoptosis was determined using an apoptosis kit based on annexin V detection (annexin V from Invitrogen). F98 cells (0.5 × 10^3^) in six-well plates were treated with 10^−5^ M of SuccFerr (as free SuccFerr or SuccFerr:RAMEβCD) for 96 h at 37 °C. Briefly, treated cells were collected, washed twice in ice-cold PBS, and then resuspended in a binding buffer at a density of 1 × 10^6^ cells/mL. The cells were incubated simultaneously with fluorescein-labeled annexin V and propidium iodide for 20 min. The mixture was then analyzed using a FACScan flow cytometer (BD Biosciences, Baltimore, MD, USA). In addition, to confirm apoptosis, apoptotic cell death was also revealed by flow cytometry using a phycoerythrin-conjugated monoclonal active caspase-3 antibody kit (BD Pharmingen, Le Pont de Claix, France), following the manufacturer’s instructions. The analysis was performed on a BD-Accuri C6 flow cytometer and data were analyzed by the FCS express 5 software.

#### 4.4.6. Hemolytic Activity

Since hemolysis is a possible effect of methylated cyclodextrins, the hemolytic activity of the compounds was tested using erythrocytes from human sources. Freshly collected blood samples were immediately mixed with an anticoagulant (Alsever’s solution, pH 7.4) to prevent blood coagulation. To obtain a pure suspension of erythrocytes, 1 mL of whole blood was diluted with 20 mL in PBS (pH 7.4) and centrifuged at 250× *g* for 5 min at 4 °C. The supernatant and buffy coats were removed by gentle aspiration, and the above process was repeated two more times. Erythrocytes were finally re-suspended in PBS to obtain a 1% solution for the hemolytic assay. For this, various concentrations of SuccFerr and SuccFerr:RAMEβCD (0.05–1 mg/mL) were added to the suspension of red blood cells. The SuccFerr- and SuccFerr:RAMEβCD-erythrocyte mixtures were incubated at 37 °C for 1 h and then centrifuged at 250× *g* for 5 min at 4 °C. The absorbance values of the supernatants were determined at 545 nm to measure the extent of red blood cell lyses. The positive controls (100% hemolysis) and negative controls (0% hemolysis) were also run by incubating erythrocytes in PBS containing 1% Triton X-100 and PBS alone, respectively [43].

### 4.5. Animal Studies

Fisher male rats were obtained from Charles River Laboratories France (L’Arbresle, France). The animals were housed in solid-bottomed plastic cages with free access to tap water and food *ad libitum*. They were maintained in standard environmental conditions (room temperature of 23 ± 1 °C, a relative humidity of 60% and a 12 h/12 h light/dark cycle).

#### 4.5.1. Animal and Anesthesia

All experiments were performed on 7 week old male Fisher rats (body weight range 170–200 g). The animals were manipulated under isoflurane/oxygen anesthesia.

#### 4.5.2. Acute Intravenous or Subcutaneously Toxicity Study in Rats

Healthy rats were randomly assigned to each of the 20 groups of 200 Fisher rats (5 females and 5 males). Rats were fasted overnight (12 h) with free access to water prior to the administration of single doses (0, 25, 50, 100, 200, 400, 800, 1600, 3200 and 6400 mg/kg) of SuccFerr:RAMEβCD dissolved in saline (NaCl 0.9%). Treatment was intraperitoneal or subcutaneous at a dose of 10 mL/kg body weight. The general behavior of the rats was continuously monitored for 4 h after the treatment, intermittently during a 24 h period [44] and, thereafter, daily up to 14 days. The LD_50_ was determined as previously described [45].

#### 4.5.3. Maximum Tolerated Dose (MTD): Sub-Acute Intraperitoneal or Subcutaneous Toxicity Study in Rats

Maximum tolerated doses (MTD) were determined to set the acceptable therapeutic doses in animal experiments. Groups of five animals (7 weeks old, 145–200 g body weight) were randomly assigned to each of eight groups of five rat males. SuccFerr:RAMβCD dissolved in saline (NaCl 0.9%) was administered subcutaneously or intravenously on every second day (48 ± 2 h) for 10 days, to groups I to IV (doses of 0, 0.5, 1 and 1.5 mg/kg, respectively). The animals were observed for signs of toxicity and mortality throughout the experimental period. The body weight, water and food consumption were recorded every 48 ± 2 h. At the end of the 10-day experiment, the animals, fasted for 12 h, were sacrificed by decapitation. None of the rats died during the experiment. Blood was collected into the following two tubes: tube 1 containing EDTA was processed immediately for hematological parameters and tube 2 without additive was centrifuged at 3000× *g* at 4 °C for 10 min to obtain serum (stored at −20 °C until analysis). The organs (kidneys, liver, lungs, heart, testes and gland annexes, ovaries, spleen and pancreas) were observed macroscopically and weighted. Organ samples (heart, kidney, pancreas, lung and liver) were either fixed in 10% formalin for histopathological examination or stored at −20 °C until biochemical analysis.

#### 4.5.4. Measurement of Hematological and Biochemical Parameters in Rats

Hematological parameters included the following: red blood cell (RBC) count, leukocyte (WBC) count, hemoglobin (Hb), hematocrit (HCt), mean corpuscular volume (MCV), mean corpuscular hemoglobin (MCH), mean corpuscular hemoglobin concentration (MCHC), platelet count, lymphocyte, monocyte, neutrophil, basophil and eosinophil counts [46]. Hematological analyses were performed using an automatic hematological analyzer (Beckman Coulter, Coulter A^c^. T BR-13692A).

For biochemical analyses, blood and urine were centrifuged at 3000 rpm for 10 min. The serum was separated and stored at −20 °C, until analytical tests could be performed. Biochemical parameters included the following: glucose, aspartate amino transferase (AST), alanine amino transferase (ALT), total cholesterol (TC), low density lipoprotein (LDL), high density lipoprotein (HDL), triglyceride (TG), alkaline phosphatase (ALP), total bilirubin (TB), conjugated bilirubin (CB), total protein (TP) and albumin (ALB). Renal function indices assay in blood was assessed by determining the concentration of creatinine, urea, uric acid, Na^+^, Cl^−^, K^+^, Ca^2+^, Mg^2+^ and inorganic phosphorus. Urine was also assessed for Na^+^, Cl^−^, K^+^, Ca^2+^, Mg^2+^, creatinine, urea and protein. The assays were made using an automatic biochemical analyzer (Hitachi 902, Roche) with a Spinreact biochemical kit (Spain).

#### 4.5.5. In Vivo Antitumor Efficacy

In vivo anticancer activity was evaluated in F98-bearing Fisher rats. Heterotopic tumor grafts were achieved as follows: after shaving and disinfection, a subcutaneous injection of 10^5^ F98 cells suspended in DMEM was performed into the right thigh. At day 15 post-tumor implantation, rats implanted with F98 cells were divided into the following three groups: group 1 was injected with physiological saline solution (NaCl 0.9%) (control; n = 8 animals), group 2 received blank RAMEβCD (n = 8 animals) and group 3 received SuccFerr:RAMEβCD (1 mg/kg; n = 8 animals). The treatment, administered via the lateral tail vein, was started on day 15 and was administered every 2 days for 10 days. Tumor size was measured 3 times in the week. The length and width of each tumor were regularly measured using a digital caliper and the tumor volume was estimated with the following mathematical ellipsoid formula: V = (π/6) × width^2^ (l) × length (L).

#### 4.5.6. Histological Analyses

At the end of the in vivo experiment (day 26), the rats were sacrificed, and the livers and kidneys were excised and immediately fixed in 4% formaldehyde solution to be enclosed in paraffin. Micrometer sections (5 µm for the liver and 2 µm for the kidney) cut with a microtome were stained with hematoxylin and eosin (H&E) for histological analyses and were examined by an anatomical pathologist.

### 4.6. Statistical Analyses

For in vitro experiments, the results were expressed as the mean ± SD and Student’s *t* test was performed between the treated and control groups. Statistical significance for the in vivo experiments was evaluated using the Mann–Whitney test and was considered as significant with *p* < 0.05. The results were expressed as a mean ± SEM.

## 5. Conclusions

In conclusion, the present investigation demonstrated that SuccFerr:RAMEßCD may be considered as relatively safe, as it did not cause any lethality nor produced any remarkable hematological, biochemical and structural adverse effects either in acute (25–100 mg/kg or 25–200 mg/kg for intravenous route) or sub-acute (0.5–2.5 mg/kg) toxicity studies in rodents. However, the significant reduction in blood ion levels and the mechanism by which liver enzyme activities decreased as observed in this study need further investigations. Consequently, the therapeutic dose was fixed at 1 mg/kg for glioblastoma treatment after this initial round of toxicity study. The in vivo antitumor activity of the SuccFerr:RAMEßCD was evaluated in the F98 tumor rat model. In vivo antitumor studies showed that repeated injections could significantly inhibit F98 tumor growth compared to the controls. This observed anticancer activity was further evidenced by a significantly reduced number of intra-tumoral proliferating cells upon SuccFerr:RAMEßCD treatment. In further studies, blood–brain barrier transport (BBB) still needs to be clarified. It is well known that pure cyclodextrins cannot cross the blood–brain barrier. In the case of complexes, cyclodextrins increase the permeability by increasing the drug solubility [47]; this molecular dispersion makes the drug available at the surface of the biological barrier. Even after the necessary dissociation, the molecular mass of SuccFerr (535 Da) can limit the BBB transport; the concept of the molecular mass threshold (400–600 Da) limiting BBB transport was advanced by Levin [48]. Nevertheless, there are occasional exceptions; for example, 20, 70 -bis(2- carboxyethyl)-5(6)-carboxyfluorescein tetraacetoxymethyl ester (BCECF-AM) has a molecular mass of 809 Da [49,50] and crosses the BBB. However, a complete pharmacokinetics study will be the next step for the development of this complex.

Finally, if the SuccFerr does not cross the BBB, our formulation could be meaningful in the treatment of breast cancer (see Figure 6; MCF7 and MDA-MB231).

## Figures and Tables

**Table 1:** Acute toxicity of SuccFerr:RAMEβCD administered by intravenous route to the rats.

Treatments	Dose (mg/kg)	D/T	Mortality Latency (h)	Toxic Symptoms
Physiological saline solution	-	0/5	-	None
SuccFerr:RAMEβCD	25	0/5	-	None
SuccFerr:RAMEβCD	50	0/5	-	None
SuccFerr:RAMEβCD	100	0/5	-	None
SuccFerr:RAMEβCD	200	2/5	>24, <36	None
SuccFerr:RAMEβCD	400	3/5	>36, <48	Hypoactivity, piloerection, salivation, asthenia
SuccFerr:RAMEβCD	800	3/5	>24, <36	Hypoactivity, piloerection, salivation, syncope
SuccFerr:RAMEβCD	1600	5/5	>24, <36	Hypoactivity, asthenia, anorexia, salivation, syncope
SuccFerr:RAMEβCD	3200	5/5	>24, <36	Hypoactivity, asthenia, anorexia, salivation, syncope
SuccFerr:RAMEβCD	6400	5/5	>24, <36	Hypoactivity, asthenia, anorexia, salivation, syncope

D/T = dead/treated rats; None = no toxic symptoms during the observation period; mortality latency = time to death (in hours) after the intravenous administration of the compound. Rats in each group were carefully examined for signs of toxic (behavioral changes and mortality) for 14 days. The control group received NaCl 0.9% (10 mL/kg, *per os*).

**Table 2:** Effects of various doses of SuccFerr:RAMEβCD administered by intravenous route on relative weights (g/100 g body weight) of organs in rats treated for 10 consecutive days.

Parameter	Vehicle	Treatments (mg/kg)		
		0.5	1	1.5
Heart	0.35 ± 0.01	0.35 ± 0.01	0.35 ± 0.01	0.35 ± 0.01
Liver	3.35 ± 0.01	3.38 ± 0.02	3.82 ± 0.04	3.84 ± 0.05
Lung	0.88 ± 0.04	0.87 ± 0.04	0.86 ± 0.03	0.85 ± 0.03
Spleen	0.72 ± 0.02	0.72 ± 0.01	0.76 ± 0.01	0.76 ± 0.01
Kidney	0.62 ± 0.01	0.66 ± 0.01	0.67 ± 0.01	0.68 ± 0.01

Values are expressed as mean ± SEM of 10 animals (5/sex). No significant difference using one way ANOVA, followed by Tukey’s multiple comparison test.

**Table 3:** Effect of sub-chronic doses of complexed SuccFerr (SuccFerr:RAMEβCD) administered by intravenous route on hematological parameters of rats.

Parameters	Treatments (mg/kg)			
	Vehicle	0.5	1	1.5
RBC (×10^6^ µL^−1^)	6.62 ± 0.13	6.35 ± 0.13	6.36 ± 0.17	5.32 ± 0.11
Hemoglobin (g/dL)	11.43 ± 0.12	11.58 ± 0.15	11.37 ± 0.92	9.35 ± 0.33
Hematocrit (%)	37.02 ± 0.16	38.19 ± 1.04	41.59 ± 0.79	37.22 ± 0.04
MCV (fL)	58.28 ± 0.35	58.76 ± 0.54	58.50 ± 0.53	57.68 ± 1.03
MCH (pg)	17.49 ± 0.15	17.49 ± 0.21	17.60 ± 0.19	17.60 ± 0.19
MCHC (g/dL)	29.56 ± 0.16	30.39 ± 0.22	30.51 ± 0.22	30.59 ± 0.14
Platelets (×10^3^ µL^−1^)	466.20 ± 7.44	456.60 ± 14.08	473.60 ± 9.28	421.40 ± 7.12
WBC (×10^3^ µL^−1^)	13.49 ± 0.21	15.46 ± 0.45	11.32 ± 0.24	9.76 ± 0.47
Neutrophils (%)	22.66 ± 0.34	24.75 ± 1.75	20.62 ± 0.46	20.47 ± 0.24
Eosinophils (%)	2.54 ± 0.14	3.25 ± 0.18	2.55 ± 0.17	2.75 ± 0.08
Basophils (%)	0.00 ± 0.00	0.00 ± 0.00	0.00 ± 0.00	0.00 ± 0.00
Lymphocytes (%)	68.88 ± 0.29	67.18 ± 1.12	65.14 ± 0.87	66.24 ± 0.26
Monocytes (%)	5.68 ± 0.18	6.34 ± 0.14	5.48 ± 0.25	5.18 ± 0.25

**Table 4:** Liver function indexes in rats administered by intravenous route with complexed SuccFerr (SuccFerr:RAMEβCD) on every second day (48 ± 2 h) for 10 days.

Parameters	Treatments (mg/kg)			
	Vehicle	0.5	1	1.5
Glu (g/L)	0.58 ± 0.03	0.62 ± 0.05	0.62 ± 0.01	0.58 ± 0.03
AST (IU/L)	217.24 ± 1.55	237.44 ± 8.13	269.30 ± 8.28 *	269.96 ± 18.85 *
ALT (IU/L)	51.72 ± 0.15	55.28 ± 0.98	55.16 ± 0.38	66.12 ± 1.18 *
ALP (IU/L)	338.14 ± 0.54	337.78 ± 0.61	323.32 ± 3.68	301.64 ± 5.76 *
TP (g/L)	71.92 ± 0.61	68.24 ± 0.59	72.74 ± 0.76	73.78 ± 0.62
ALB (g/L)	21.28 ± 0.15	23.80 ± 0.17	24.2 ± 0.13	24.12 ± 0.19
TB (mg/L)	6.56 ± 0.43	7.24 ± 0.43	7.68 ± 0.66	7.86 ± 0.80
CB (mg/L)	3.76 ± 0.25	4.44 ± 0.37	4.68 ± 0.18	5.66 ± 0.37
TC (g/L)	0.60 ± 0.02	0.61 ± 0.01	0.61 ± 0.01	0.62 ± 0.04
LDL (g/L)	0.19 ± 0.01	0.19 ± 0.01	0.19 ± 0.02	0.19 ± 0.01
HDL (g/L)	0.538 ± 0.020	0.53 ± 0.02	0.53 ± 0.02	0.54 ± 0.02
TG (g/L)	1.43 ± 0.13	1.63 ± 0.09	1.75 ± 0.06	1.63 ± 0.03

Values are mean ± SEM of 5 male rats. Data were analyzed by two-way ANOVA, followed by Tukey’s multiple comparison test. * *p* < 0.05 significantly different from the control. Glu: glucose, ALT: alanine amino transferase, AST: aspartate amino transferase, ALP: alkaline phosphatase, ALB: albumin, TP: total protein, TB: total bilirubin, CB: conjugated bilirubin, TG: triglyceride, TC: total cholesterol, LDL: low-density lipoprotein, HDL: high-density lipoprotein.

**Table 5:** Renal function measures from blood of rats administered by intravenous route with complexed SuccFerr (SuccFerr:RAMEβCD) on every second day (48 ± 2 h) for 10 days.

Parameters	Treatments (mg/kg)			
	Vehicle	0.5	1	1.5
Creatinine (mg/L)	7.32 ± 0.26	8.94 ± 0.31	8.88 ± 0.19	7.35 ± 0.27
Urea (mg/L)	0.422 ± 0.010	0.39 ± 0.01	0.39 ± 0.05	0.425 ± 0.020
Uric acid (mg/L)	46.92 ± 0.52	45.88 ± 0.73	43.66 ± 1.19	46.90 ± 0.50
Na^+^ (mEquiv./L)	134.72 ± 0.51	134.24 ± 1.29	131.06 ± 2.59	134.76 ± 0.50
Cl^−^ (mEquiv./L)	85.64 ± 1.03	85.74 ± 1.74	86.72 ± 1.08	84.46 ± 1.51
K^+^ (mEquiv./L)	3.44 ± 0.21	3.64 ± 0.18	3.36 ± 0.23	3.42 ± 0.25
Ca^2+^ (mg/L)	77.94 ± 0.47	78.52 ± 0.42	69.22 ± 1.14	67.04 ± 1.51
Mg^2+^ (mg/L)	17.87 ± 0.16	19.28 ± 0.47	18.19 ± 0.64	19.55 ± 0.67
Inorganic phosphorus (mg/L)	33.08 ± 1.37	30.52 ± 1.32	29.58 ± 0.65	29.18 ± 1.96

Values are mean ± SEM of 5 male rats. Data were analyzed by two-way ANOVA, followed by Tukey’s multiple comparison test. There were no significant differences compared to the control group.

## Data Availability

All data used to support the findings of this study are included within the article.

## Supplementary Materials

The following supporting information can be downloaded at: https://www.mdpi.com/article/10.3390/molecules27144651/s1, Figure S1: Representative histopathology images of kidneys (panels A-B) and liver (panels C-D) obtained from rats used for the in vivo evaluation of SuccFerr:RAMEβCD anticancer activity in a murine glioblastoma model, and treated during 10 days with i.v. injections of SuccFerr:RAMEβCD (1 mg/kg). A qualified senior pathologist (N.S.) did not observe any sign of toxicity in the kidneys and the livers of the animals treated by SuccFerr:RAMEβCD, when compared to the control group.

## Sample Availability

Samples of the compounds are not available from the authors.
