# Supplementary material for: Succinimido–Ferrocidiphenol Complexed with Cyclodextrins Inhibits Glioblastoma Tumor Growth In Vitro and In Vivo without Noticeable Adverse Toxicity
Source: Molecules. 2022 Jul 21;27(14):4651. doi: 10.3390/molecules27144651 (PMC9316017; doi:10.3390/molecules27144651)

Supplementary Figure S1

Representative histopathology images of kidneys (panels A-B) and liver (panels C-D) obtained from rats used for the *in vivo* evaluation of SuccFerr:RAMEβCD anticancer activity in a murine glioblastoma model, and treated during 10 days with i.v. injections of SuccFerr:RAMEβCD (1 mg/kg). A qualified senior pathologist (N.S.) did not observe any sign of toxicity in the kidneys and the livers of the animals treated by SuccFerr:RAMEβCD, when compared to the control group.

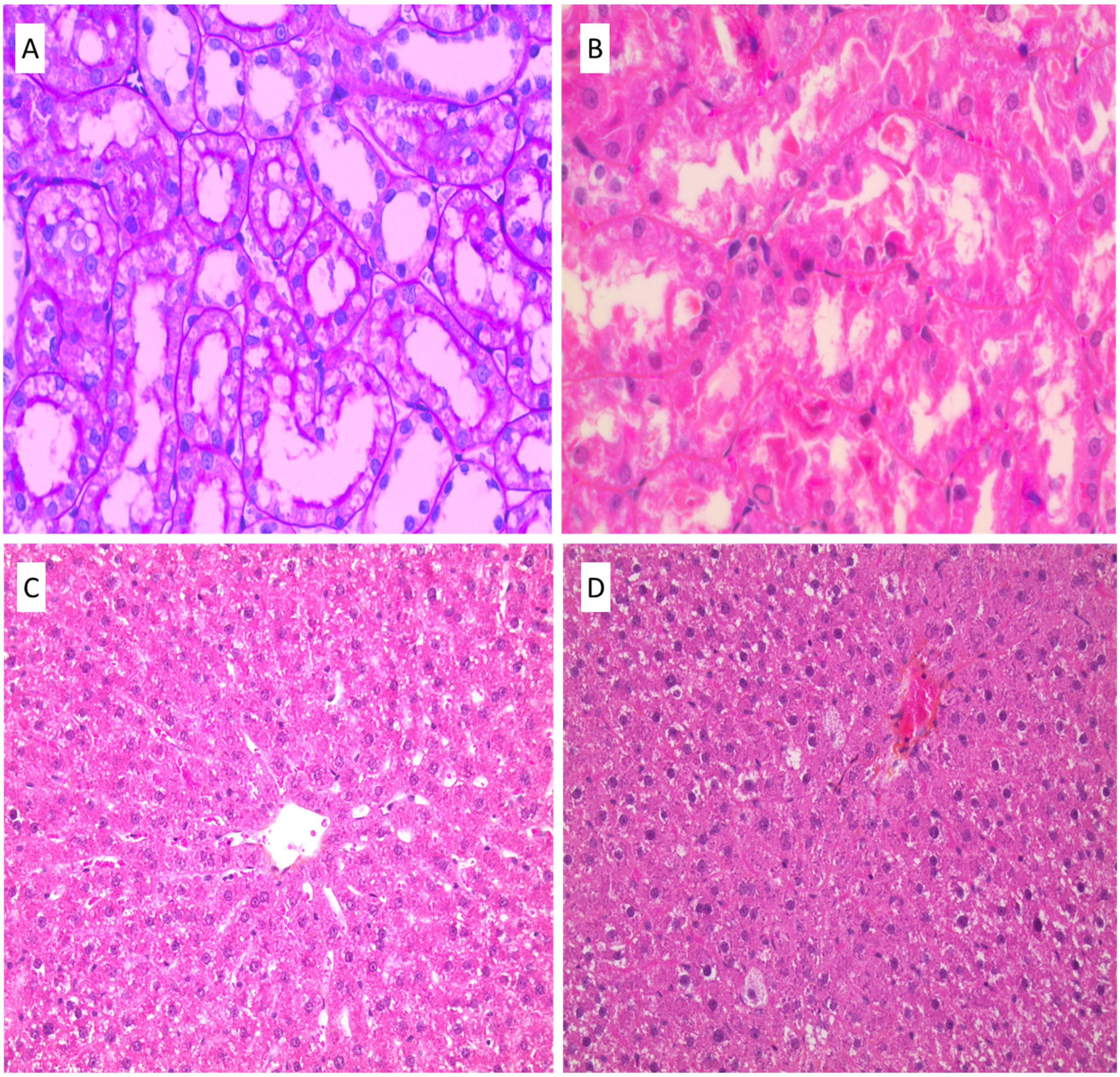

Supplement: Supplementary file 1 [file molecules-27-04651-s001.zip › molecules-1796180-supplementary.pdf]
